# Evaluation of Transmission Parameters of Three Korean African Swine Fever Virus Isolates: A Bayesian ABC‐SMC and Agent‐Based Modeling Approach

**DOI:** 10.1155/tbed/4377003

**Published:** 2026-11-01

**Authors:** Ki-Hyun Cho, Da-Young Kim, Yong-Myung Kang, Yeon-Hee Kim, Hae-Eun Kang, Dae Sung Yoo

**Affiliations:** ^1^ Foreign Animal Disease Division Animal and Plant Quarantine Agency 177 Hyeoksin 8-ro Gimcheon 39660 Gyeongsangbuk-do Republic of Korea qia.go.kr; ^2^ College of Veterinary Medicine Kyungpook National University 80 Daehak-ro, Buk-gu Daegu 41566 Republic of Korea knu.ac.kr; ^3^ College of Veterinary Medicine Chonnam National University 77 Yongbong-ro, Buk-gu Gwangju 61186 Republic of Korea jnu.ac.kr

## Abstract

African swine fever (ASF) remains a major threat to global pig production. An understanding of within‐pen transmission dynamics is essential for designing effective prevention and control. Three independent direct‐contact (DC) transmission experiments were conducted using Korean ASF virus (ASFV) isolates representing distinct epizootic phases in the Republic of Korea: Korea/Pig/Paju1/2019, Korea/Pig/Yeongwol/2021, and Korea/Pig/Hongcheon/2023. In each experiment, two inoculated donor pigs were cohoused with five DC‐susceptible pigs for up to 18 days postinoculation (dpi). Transmission parameters were estimated using two complementary modeling approaches. First, an approximate Bayesian computation sequential Monte Carlo (ABC‐SMC) framework was applied to a deterministic susceptible–exposed–infectious–removed (SEIR) ordinary differential equation (ODE) model. Second, a stochastic agent‐based model (ABM) was used to validate parameter estimates and quantify within‐pen outbreak probability. All DC pigs in the three experimental groups developed ASFV infection and died within 18 days postexposure. The Bayesian SEIR model estimated transmission coefficients (*β*) of 0.503, 0.435, and 0.308 per susceptible–infectious pair per day for Paju, Yeongwol, and Hongcheon isolates, respectively. Notably, the Korea/Pig/Hongcheon/2023 isolate exhibited a shorter posterior latent period (*ε* = 3.78 days; 95% credible interval [CrI]: 2.78–4.83) than Korea/Pig/Paju1/2019 (*ε* = 7.23 days; 95% CrI: 5.36–9.48) and Korea/Pig/Yeongwol/2021 (*ε* = 6.11 days; 95% CrI: 4.57–7.73). ABM simulations using posterior parameter distributions yielded per‐donor secondary infection estimates (*R*_pd_; the mean number of DC pigs infected per‐donor pig, structurally bounded by the pen size) of 1.95, 2.10, and 2.37 for the Paju, Yeongwol, and Hongcheon isolates, respectively. In sensitivity analyses, the shorter latent period of the Hongcheon/2023 isolate was consistently observed under alternative exposure‐time assumptions, distance‐function weightings, and prior specifications, although the absolute values of *β* and *ε* depended on the assumed exposure time. This study provides the first comparative analysis of within‐pen transmission dynamics for multiple Korean ASFV isolates. Although overall transmission potential was similar across isolates, the Korea/Pig/Hongcheon/2023 isolate exhibited a shorter estimated latent period and a marginally higher *R*_pd_. Because isolate identity was confounded with experiment year, this difference requires confirmation in contemporaneous experiments; nevertheless, it may have implications for early detection and rapid spread within pig facilities.

## 1. Introduction

African swine fever (ASF) is a major infectious disease of swine characterized by severe febrile and hemorrhagic clinical signs [1]. As a notifiable disease designated by the World Organization for Animal Health (WOAH), ASF restricts international trade in pigs and pig products and causes substantial economic losses to national economies and the swine industry [2–4]. Live attenuated ASF vaccines have recently been licensed and deployed in a small number of countries, most notably Vietnam [5, 6]; however, no vaccine is yet widely available in most affected regions, including the Republic of Korea, and no effective treatment exists. Therefore, early detection, rapid culling, and strict movement control remain the primary strategies for disease control [7, 8].

Over the past 18 years, ASF has spread extensively and has now been reported on every continent except Antarctica. In Asia, the epidemiological situation of ASF has progressively worsened following the initial incursion of ASF into China in 2018. In the Republic of Korea, ASF was first confirmed in September 2019 at a pig farm in Paju, Gyeonggi Province, which subsequently spread to additional farms and wild boar populations [9, 10]. Epidemic analyses identified at least three genetically distinct waves associated with different Korean ASF virus (ASFV) isolates recovered in 2019, 2021, and 2023, providing a unique opportunity to compare the virological and epidemiological characteristics of viruses from different epizootic phases.

A clear understanding of transmission dynamics within pig pens is essential for evaluating the effectiveness of disease control and preventive strategies. Quantitative estimates of transmission parameters provide critical insights into the epidemiological behavior of ASFV, including the basic reproduction number (*R*_0_), transmission coefficient (*β*), and latent and infectious‐period durations. These parameters inform the expected speed of pen‐level spread, proportion of a herd at risk, and time window available for detection and intervention [11–14].

Several animal transmission experiments using ASFV isolates have been conducted globally. Guinat et al. [15] estimated *R*_0_ = 2.5 (95% confidence interval [CI]: 1.3–4.8) for the Georgia 2007/1 genotype II strain using a stochastic susceptible–exposed–infectious–removed (SEIR) model in a controlled pen. Main et al. [16] applied Bayesian inference to estimate the transmission dynamics for European ASFV and reported within‐pen *R*_0_ values ranging from 1.8 to 4.6. More recently, Oh et al. [17] estimated *R*_0_ values of 2.916 and 4.015 for a Vietnamese ASFV isolate using exponential growth and maximum likelihood methods, respectively. Although these studies provide valuable reference values, the transmission parameters for Korean ASFV isolates from different outbreak periods have not been systematically compared.

Several animal experiments using Korean ASFV isolates have been conducted to evaluate viral virulence and compare disease progression according to the different transmission routes [18–20]. These investigations focused primarily on clinical and pathological outcomes but did not quantify within‐pen transmission dynamics using mathematical modeling approaches. In particular, a comparative analysis of transmission parameters across Korean ASFV isolates isolated in different years, representing distinct phases of the Korean epidemic, has not been conducted.

Accordingly, this study aimed to estimate and compare within‐pen transmission parameters for three Korean ASFV isolates—Korea/Pig/Paju1/2019, Korea/Pig/Yeongwol/2021, and Korea/Pig/Hongcheon/2023—using two complementary modeling approaches: an approximate Bayesian computation sequential Monte Carlo (ABC‐SMC) framework applied to a deterministic SEIR model and a stochastic agent‐based model (ABM). To the best of our knowledge, this study provides the first comparative analysis of ASFV transmission dynamics across Korean ASFV isolates from different epidemic periods, offering insights into potential changes in the epidemiological behavior of ASFV circulating in the Republic of Korea.

## 2. Materials and Methods

### 2.1. Virus Isolation

Of the 28 ASFV isolates obtained from domestic pig farms in South Korea between 2019 and 2023, three were selected for this study based on genetic analysis and epidemiological relevance. The three isolates were designated Korea/Pig/Paju1/2019 (group 1), Korea/Pig/Yeongwol/2021 (group 2), and Korea/Pig/Hongcheon/2023 (group 3). All three strains belonged to ASFV genotype II and were isolated from the spleens of dead pigs at the affected farms. Pulmonary alveolar macrophage (PAM) cells (Optipharm, South Korea) were used for virus isolation and propagation. Spleen samples were homogenized in sterile phosphate‐buffered saline, filtered, and inoculated onto PAM cell monolayers. Propagated viruses were titrated using the hemadsorption assay, and each isolate was standardized to 10^3^ HAD_50_/mL for experimental inoculation.

### 2.2. Animal Experiments and Sample Collection

Three independent animal experiments were performed using a total of 27 8‐week‐old Landrace pigs obtained from a commercial pig farm with no reported history of major swine viral diseases, including ASF, classical swine fever, foot‐and‐mouth disease, and porcine reproductive and respiratory syndrome. Each experiment comprised two inoculated donor pigs, five direct‐contact (DC) pigs, and two noncontact control pigs, all housed in a biosafety level 3 (BSL‐3) facility at the Animal and Plant Quarantine Agency, Republic of Korea. Donor pigs were inoculated intramuscularly with 10^3^ HAD_50_ of the respective ASFV isolate on day‐0 postinoculation (dpi). DC pigs were cohoused with donors in the same pen from day 0, without physical separation from the donor animals.

Blood samples, oral swabs, and rectal swabs were collected daily from all pigs. ASFV DNA was detected by real‐time polymerase chain reaction (qPCR) targeting the *p72* gene, following the WOAH‐recommended protocol [21]. Viral load was quantified as log_10_ genome copies per reaction (log_10_ copies/µL) using a standard curve derived from a plasmid containing the *p72* gene fragment. Viremia was defined as the first day of the detection of ASFV DNA in whole blood samples. Infectious viral‐shedding onset was defined as the first day of detection of ASFV DNA in oral or rectal swab samples (whichever occurred first). All pigs were monitored daily until natural death or humane euthanasia as per the institutional animal ethics guidelines.

### 2.3. Clinical Assessment

Clinical observations were recorded daily for each pig until death or the humane endpoint using a clinical scoring system [22]. The scoring scheme ranges from 0 to 40 points and comprises 10 clinical parameters, such as body temperature, activity level, respiratory signs, and neurological signs. Rectal body temperature was recorded daily; pyrexia was defined as a rectal temperature exceeding 40°C. Animals were euthanized when clinical scores reached a predefined humane endpoint or upon the observation of terminal clinical signs.

### 2.4. Estimation of Transmission Parameters

#### 2.4.1. SEIR Model Structure

Transmission dynamics within each experimental pen was modeled using a deterministic SEIR compartmental framework. The model assumes mass‐action (density‐dependent) transmission and describes the transitions between compartments using the following system of ordinary differential equations (ODEs):

dSdt=−β⋅S⋅I,

dE/dt=β⋅S⋅I−E/ε,

dIdt=E/ε−I/r,

dRdt=Ir,

where *S*, *E*, *I*, and *R* denote the number of pigs in the susceptible, exposed (latent), infectious, and removed (dead) states, respectively. The parameters are as follows: *β*, transmission coefficient (per susceptible–infectious pair per day); *ε*, mean latent period (days), representing the average time from exposure to the onset of viral shedding; and *r*, mean infectious period (days), representing the average time from the onset of shedding to death. The basic reproduction number was calculated as *R*_0_ = *β*·*S*_0_·*r*, where *S*_0_ = 5 is the initial number of susceptible DC pigs in each experiment.

Initial conditions were set as *S* (0) = 5, *E* (0) = 2 (representing two donor pigs in the latent phase immediately following inoculation), *I* (0) = 0, and *R* (0) = 0. SEIR state time series were reconstructed from the experimental data as follows: a pig was classified as being in state *I* from the first day of detectable viral shedding (positive oral or rectal swab by qPCR) until death (transition to *R*). Donor pigs were assumed to enter the *E* state at day 0 (inoculation day), consistent with the model initial conditions. DC pigs were assumed to enter the *E* state when donor pigs first began shedding virus (day 4 in all experiments, based on observed donor shedding onset) and remained in *E* until their shedding onset. This assumption was adopted because oronasal shedding is considered the principal source of contact infectiousness in DC settings, making donor shedding onset the earliest biologically plausible time of effective exposure. We acknowledge, however, that true infection times of DC pigs are latent and unobserved; this reconstruction therefore encodes an a priori assumption rather than an observed quantity, and all event times (exposure, shedding onset, and death) are interval‐censored at the daily sampling resolution. The influence of the assumed exposure time and of the daily temporal resolution on the posterior estimates was formally examined through the sensitivity analyses described in Section 2.4.4. ODE integration was performed using the scipy.integrate.odeint solver (Python 3.10 and SciPy 1.11).

#### 2.4.2. Bayesian ABC‐SMC

Bayesian parameter inference was performed using the ABC‐SMC algorithm as implemented in the pyabc library (Python 3.10) [23]. ABC‐SMC is particularly suited to models in which the likelihood function is computationally intractable, as is the case for differential equation models fitted to small‐sample trajectory data. Although a likelihood‐based formulation (e.g., a chain‐binomial or transition‐time survival likelihood) would be feasible if all transition times were fully and exactly observed, the latent‐state trajectories in this study are partially reconstructed and interval‐censored. The simulation‐based ABC‐SMC framework was therefore preferred because it requires only forward simulation of the model, readily accommodates alternative assumptions regarding unobserved processes such as exposure timing, and provides finite‐sample posterior uncertainty statements for all parameters simultaneously. The algorithm iteratively refines a population of parameter particles toward the posterior distribution by progressively lowering an acceptance threshold (tolerance; distinct from the latent period *ε*) over sequential generations.

Prior distributions were specified based on published literature on ASFV infection dynamics and expert knowledge: for *β*, Uniform(0, 3) per pair per day (uninformative, bounded by biological plausibility); for *ε*, a gamma distribution with shape 4 and scale 1 shifted by 1 day (Gamma(4, 1) + 1; prior mean 5 days; reflecting reported ASFV previremic period) for the Paju1/2019 and Yeongwol/2021 isolates and Gamma(3, 1) + 1 (prior mean 4 days) for the Hongcheon/2023 isolate, reflecting the earlier onset of viral shedding observed in its DC pigs; and for *r*, Gamma(5, 1) + 1 (prior mean 6 days; reflecting observed postshedding survival duration in DC pigs). Because the Hongcheon/2023 latent‐period prior was informed by observations from the same experiment, its influence was examined by refitting this isolate with the common Gamma(4, 1) + 1 prior (Section 2.4.4). The distance function compared simulated and observed daily counts in the *E*, *I*, and *R* compartments as the weighted sum over compartments of the square root of the summed absolute differences across days, and weights of 1.0, 2.0, and 1.5 were assigned for *E*, *I*, and *R*, respectively, to emphasize the infectious compartment fit. The *I* compartment received the largest weight because infectious‐state occupancy is the most directly observed quantity (qPCR‐confirmed shedding), whereas the reconstructed *E* compartment partly reflects the assumed exposure time; the *R* compartment (death) is directly observed but conveys partially redundant information with *I*. The robustness of the posterior estimates to this weighting scheme, including equal weights and complete exclusion of the *E* compartment from the distance function, was assessed in sensitivity analyses (Section 2.4.4). Given the small number of animals per experiment (five DC pigs), we also assessed prior sensitivity and note that the joint identifiability of *β*, *ε*, and *r* is limited by the information content of a single small‐pen epidemic; posterior credible intervals (CrIs) should be interpreted accordingly. Each ABC‐SMC run adopted a population size of 800 particles and ran for up to six sequential generations or until the acceptance tolerance reached 0.5. Analyses were conducted independently for each of the three isolates. Posterior samples were summarized by the weighted median and 95% CrIs derived from weighted quantiles. Posterior resampling and ABM simulations (Section 2.4.3) used fixed random seeds for reproducibility; the ABC‐SMC runs were not seeded, so repeated runs yield slightly different posterior summaries owing to Monte Carlo variability.

#### 2.4.3. Stochastic ABM

A stochastic ABM was developed to (i) validate the Bayesian SEIR parameter estimates by simulating epidemic trajectories under posterior uncertainty and (ii) derive a per‐donor secondary infection estimate (*R*_pd_). In the ABM, each pig is represented as an individual agent with a discrete state (*S*, *E*, *I*, or *R*). State transitions follow Bernoulli processes at each daily time step (*dt* = 1 day): the probability of *S*→*E* for each susceptible pig is 1 − exp(−*β* · It), where It is the number of currently infectious pigs on day *t*; the probability of *E*→*I* is 1 − exp(− 1/*ε*); and the probability of *I*→*R* is 1 − exp(− 1/*r*). These Bernoulli probabilities are derived from the exponential holding‐time assumption underlying the deterministic SEIR model and ensure consistency between the two approaches.

For each isolate, 2000 ABM simulations were run, with each simulation drawing a parameter set (*β*, *ε*, *r*) by weighted random sampling from the ABC‐SMC posterior distribution. Initial conditions matched those of the SEIR model (*S* = 5 DC pigs and *E* = 2 donors). *R*_pd_ was estimated from each simulation as the total number of DC pigs infected (in states *I* or *R* at the simulation end) divided by 2 (the number of donor pigs). Because this quantity is structurally bounded above by 2.5 (five DC pigs per two donors), it should be interpreted as a per‐donor final infection ratio over the experimental period rather than as a true basic reproduction number in the classical sense. Fan charts of simulated *I*(*t*) trajectories (50th, 25th–75th, and 2.5th–97.5th percentile prediction intervals [PIs]) were plotted alongside observed daily *I*(*t*) counts to assess the model fit. The probability of complete pen‐level spread (all five DC pigs becoming infected) was estimated as the proportion of simulations in which all five DC pigs transitioned out of the *S* state.

#### 2.4.4. Sensitivity Analyses

Because the exposure times of DC pigs were fixed a priori and the ABC distance function involves user‐specified choices, we conducted a structured set of sensitivity analyses in which the ABC‐SMC inference was repeated for each isolate under alternative specifications, holding all other settings (population size, number of generations, and stopping rule) identical to those in the baseline analysis. Three groups of alternatives were examined. First, for exposure timing, the observed state trajectories were reconstructed assuming that all DC pigs entered the *E* compartment at day 0 (start of cohousing, the earliest possible exposure) or at day 3 (donor viremia onset), instead of day 4 (donor shedding onset, baseline); because the transmission model itself is unchanged, these scenarios bound the plausible range of exposure times. Second, for the distance function, we considered equal compartment weights (*E* = *I* = *R* = 1.0) and complete exclusion of the reconstructed *E* compartment from the distance (weights *E* = 0, *I* = 2.0, and *R* = 1.5), the latter removing any direct influence of the exposure‐time assumption on the fitting target. Third, for prior specification, we replaced the baseline priors with vague uniform priors (*ε*, *r* ~ Uniform(1, 16) days) and, for the Hongcheon/2023 isolate, with the same Gamma(4, 1) + 1 latent‐period prior used for the other two isolates (common prior), to verify that its shorter estimated latent period was not induced by its baseline Gamma(3, 1) + 1 prior. For comparability, the baseline specification was also rerun alongside the sensitivity scenarios; all scenarios in Supporting Information 1: Table S1 are compared with this rerun baseline, whose estimates differ slightly from the primary estimates in Table 1 owing to Monte Carlo variability. Posterior medians and 95% CrIs for all scenarios are reported in Supporting Information 1: Table S1 and visualized in Supporting Information 2: Figure S3.

**Table 1:** Summary of Bayesian ABC‐SMC posterior estimates for SEIR transmission parameters.

Group (isolate)	*β* (day^−1^) median (95% CrI)	*ε* (days) median (95% CrI)	*r* (days) median (95% CrI)	*R*_0_ (SEIR) median (95% CrI)	*R*_pd_ (ABM) mean ± SD (95% PI)
Group 1 (Korea/Pig/Paju1/2019)	0.503 (0.209–1.071)	7.23 (5.36–9.48)	8.23 (6.41–11.10)	20.18 (8.61–50.62)	1.95 ± 0.58 (0.50–2.50)
Group 2 (Korea/Pig/Yeongwol/2021)	0.435 (0.216–0.799)	6.11 (4.57–7.73)	6.86 (5.43–8.97)	14.66 (7.92–29.60)	2.10 ± 0.50 (1.00–2.50)
Group 3 (Korea/Pig/Hongcheon/2023)	0.308 (0.180–0.498)	3.78 (2.78–4.83)^a^	7.68 (5.78–10.66)	11.83 (6.80–20.53)	2.37 ± 0.32 (1.50–2.50)

*Note:* Values are posterior medians with 95% CrI for the transmission coefficient (*β*), latent period (*ε*), infectious period (*r*), and within‐pen basic reproduction number (*R*_0_ = *β*·*S*_0_·*r*) for three Korean ASFV isolates. The final column reports the ABM‐derived per‐donor secondary infection estimate (*R*_pd_; mean ± SD with 95% prediction interval), which is structurally bounded above by 2.5 (five DC pigs per two donors) and should not be interpreted as a basic reproduction number.

Abbreviations: ABC‐SMC, approximate Bayesian computation sequential Monte Carlo; CrI, credible interval; SEIR, susceptible–exposed–infectious–removed.

^a^Shorter than Groups 1 and 2, with nonoverlapping 95% CrIs.

## 3. Results

### 3.1. Clinical Assessment and Disease Dynamics

Across all three experimental groups, donor pigs exhibited comparable disease dynamics. Viremia was first detected at a mean of 3.0–3.5 days postinoculation (dpi), followed by the onset of pyrexia (>40°C) at 4.0–6.0 dpi. Viral shedding through oral and rectal routes was detected 1.0–2.0 days after viremia onset. The mean survival period of donor pigs was 7.0 dpi in group 1 (Paju), 9.0 dpi in group 2 (Yeongwol), and 9.5 dpi in group 3 (Hongcheon), indicating slightly longer survival in later‐year isolates.

In group 1 (Paju), DC pigs became viremic at 9.8 ± 0.4 dpi and developed fever (>40°C) from 11.6 ± 0.9 dpi. Death occurred at 16.6 ± 1.5 dpi. Continuous viral excretion via oral and rectal routes was detected at 10.8 ± 0.4 dpi and 11.6 ± 1.3 dpi, respectively. In group 2 (Yeongwol), DC pigs began shedding virus from 10.4 ± 1.1 dpi (rectal) and died at 15.2 ± 1.8 dpi. In group 3 (Hongcheon), DC pigs showed the earliest viral‐shedding onset (rectal shedding from 8.8 ± 0.8 dpi) and the fastest progression to death (13.6 ± 0.9 dpi). All five DC pigs in each of the three experiments developed ASFV infection and died or reached the humane endpoint within the observation period, indicating a 100% attack rate in all groups. The clinical disease course is summarized in Table 2 and illustrated in Figure 1.

**Figure 1:**
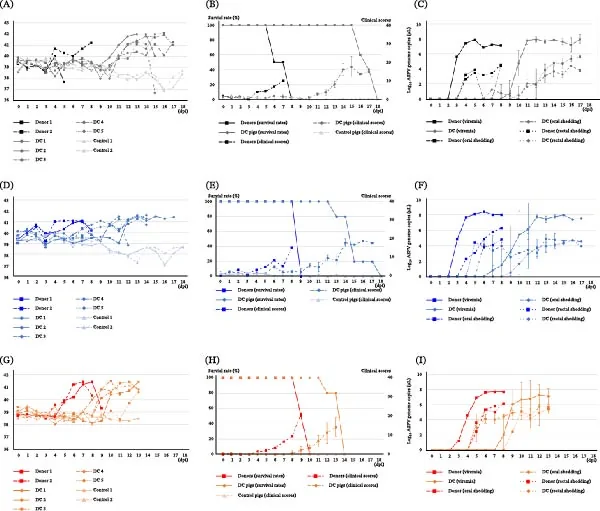
Clinical disease course of donors and direct‐contact pigs in three groups. In each group, two donor pigs were challenged with respective Korean virus strains (Korea/Pig/Paju1/2019, Korea/Pig/Yeongwol/2021, and Korea/Pig/Hongcheon/2023). (A) Rectal temperature in group 1. (B) Survival rates and clinical scores in group 1. (C) Viremia and viral shedding in group 1. (D) Rectal temperature in group 2. (E) Survival rates and clinical scores in group 2. (F) Viremia and viral shedding in group 2. (G) Rectal temperature in group 3. (H) Survival rates and clinical scores in group 3. (I) Viremia and viral shedding in group 3.

**Table 2:** Transmission characteristics of donor and direct‐contact pigs in three experimental groups.

Group (inoculated ASFV isolate)	Donor pigs (*n* = 2), mean					Direct contact pigs (*n* = 5), mean ± standard deviation				
	Survival period	Onset of fever	Onset of viremia	Onset of oral shedding	Onset of rectal shedding	Survival period	Onset of fever	Onset of viremia	Onset of oral shedding	Onset of rectal shedding
1 (Korea/Pig/Paju1/2019)	7.0	4.0	3.0	4.0	5.0	16.6 ± 1.5	11.6 ± 0.9	9.8 ± 0.4	10.8 ± 0.4	11.6 ± 1.3
2 (Korea/Pig/Yeongwol/2021)	9.0	4.0	3.0	4.0	5.0	15.2 ± 1.8	9.4 ± 1.9	9.4 ± 1.8	6.0 ± 0	10.4 ± 1.1
3 (Korea/Pig/Hongcheon/2023)	9.5	6.0	3.5	5.0	5.0	13.6 ± 0.9	9.8 ± 1.9	8.6 ± 0.5	5.2 ± 0.4	8.8 ± 0.8

*Note:* Survival period, onset of fever, viremia, oral shedding, and rectal shedding are presented as mean ± SD (days postinoculation).

### 3.2. Bayesian ABC‐SMC Estimation of SEIR Parameters

The Bayesian ABC‐SMC analysis yielded posterior distributions for the three SEIR parameters (*β*, *ε*, and *r*) for each isolate. Table 1 summarizes the posterior medians and 95% CrIs; Figure 2 displays the posterior probability density functions for all three parameters across the three isolates.

**Figure 2:**
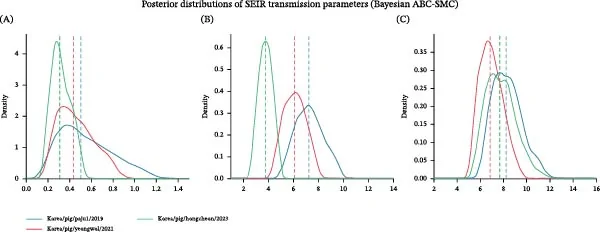
Posterior probability density functions of SEIR transmission parameters estimated by Bayesian ABC‐SMC for three Korean ASFV isolates. The dashed vertical lines indicate posterior medians. (A) Transmission coefficient (*β*, per susceptible–infectious pair per day); (B) mean latent period (*ε*, days); and (C) mean infectious period (*r*, days). ABC‐SMC, approximate Bayesian computation sequential Monte Carlo; ABM, agent‐based model; ASFV, African swine fever virus; SEIR, susceptible–exposed–infectious–removed.

For the Paju1/2019 isolate, the posterior transmission coefficient was *β* = 0.503 (95% CrI: 0.209–1.071) per susceptible–infectious pair per day. The estimated mean latent period was *ε* = 7.23 days (95% CrI: 5.36–9.48), and the mean infectious period was *r* = 8.23 days (95% CrI: 6.41–11.10). The within‐pen basic reproduction number was *R*_0_ = 20.18 (95% CrI: 8.61–50.62). Joint posterior scatter plots of *β* (x‐axis) versus *r* (y‐axis), obtained for each isolate using Bayesian ABC‐SMC analysis, are shown in Supporting Information 2: Figure S1.

For the Yeongwol/2021 isolate, *β* = 0.435 (95% CrI: 0.216–0.799), *ε* = 6.11 days (95% CrI: 4.57–7.73), and *r* = 6.86 days (95% CrI: 5.43–8.97). The within‐pen *R*_0_ was 14.66 (95% CrI: 7.92–29.60).

For the Hongcheon/2023 isolate, *β* = 0.308 (95% CrI: 0.180–0.498), *ε* = 3.78 days (95% CrI: 2.78–4.83), and *r* = 7.68 days (95% CrI: 5.78–10.66). The within‐pen *R*_0_ was 11.83 (95% CrI: 6.80–20.53). Notably, the latent period for the Hongcheon/2023 isolate was substantially shorter than for the Paju1/2019 and Yeongwol/2021 isolates, with nonoverlapping 95% CrIs, suggesting a difference in incubation kinetics under the shared exposure‐time assumption applied to all three experiments (Section 3.4 for sensitivity analyses). Posterior distributions for the latent and infectious periods are presented in Figure 3.

**Figure 3:**
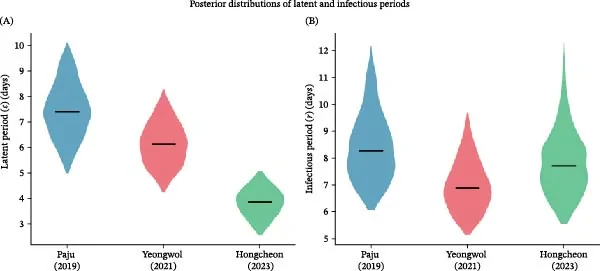
Posterior distributions of (A) latent period (*ε*) and (B) infectious period (*r*) for three Korean ASFV isolates, displayed as violin plots. The black horizontal bars indicate posterior medians. The latent period of Korea/Pig/Hongcheon/2023 is substantially shorter than those of the 2019 and 2021 isolates, with nonoverlapping 95% credible intervals. ASFV, African swine fever virus.

### 3.3. Stochastic ABM Simulations

Figure 4 presents the observed daily infectious pig counts *I*(*t*) overlaid with the ABM PIs derived from 2000 simulations per isolate. The ABM fan charts encompassed the observed trajectories across all three experimental groups, indicating adequate model fit despite the small experimental pen sizes.

**Figure 4:**
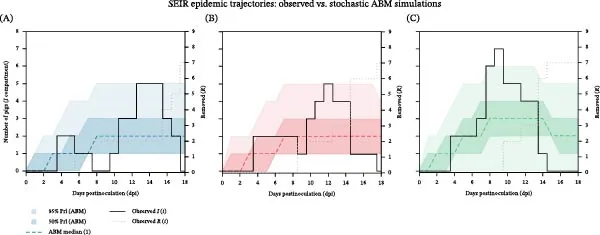
Epidemic trajectories of the SEIR infectious compartment *I*(*t*) for three Korean ASFV isolates. The black step lines represent the observed daily counts of infectious (viral‐shedding) pigs. The shaded bands indicate 50% (dark) and 95% (light) stochastic prediction intervals from 2000 ABM simulations using parameters drawn from Bayesian ABC‐SMC posterior distributions. The gray dotted lines represent cumulative removed (dead) pigs *R*(*t*). (A) Korea/Pig/Paju1/2019; (B) Korea/Pig/Yeongwol/2021; and (C) Korea/Pig/Hongcheon/2023. ABC‐SMC, approximate Bayesian computation sequential Monte Carlo; ABM, agent‐based model; ASFV, African swine fever virus; SEIR, susceptible–exposed–infectious–removed.

Per‐donor secondary infection estimates (*R*_pd_) derived from ABM simulations were 1.95 ± 0.58 (95% PI: 0.50–2.50) for Paju1/2019; 2.10 ± 0.50 (95% PI: 1.00–2.50) for Yeongwol/2021; and 2.37 ± 0.32 (95% PI: 1.50–2.50) for Hongcheon/2023. These values represent the mean number of DC pigs infected per‐donor pig during the experimental observation period and are bounded above by 2.5, given the experimental design. The *R*_pd_ was the highest for the Hongcheon/2023 isolate, consistent with its shorter latent period and earlier onset of viral shedding in DC pigs. Figure 5 compares the SEIR‐derived *R*_0_ and the ABM‐derived *R*_pd_ across the three isolates.

**Figure 5:**
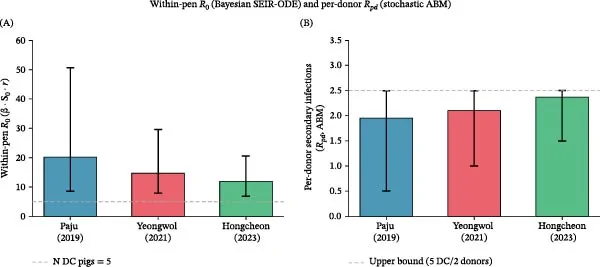
Within‐pen basic reproduction number (*R*_0_) estimates from the Bayesian SEIR‐ODE model and per‐donor secondary infection estimates (*R*_pd_) from the stochastic ABM. (A) Within‐pen *R*_0_ calculated as *β* · *S*_0_ · *r* from Bayesian SEIR posterior medians; error bars indicate 95% credible intervals. (B) *R*_pd_ from 2000 ABM simulations per isolate; error bars indicate 95% prediction intervals. The dashed reference lines indicate *S*_0_ = 5 DC pigs (A) and the structural upper bound of *R*_pd_ (five DC pigs per two donors; B). ABC‐SMC, approximate Bayesian computation sequential Monte Carlo; ABM, agent‐based model; ASFV, African swine fever virus; DC, direct‐contact; ODE, ordinary differential equations; SEIR, susceptible–exposed–infectious–removed.

The probability of complete pen‐level spread (all five DC pigs infected) across 2000 simulations was 36.4% for Paju1/2019, 48.9% for Yeongwol/2021, and 81.0% for Hongcheon/2023 (Supporting Information 2: Figure S2). The higher probability of complete spread for Hongcheon/2023 relative to the 2019 and 2021 isolates is largely attributable to its shorter latent period, which accelerates the infectious‐state transition and increases the cumulative probability that all DC pigs become exposed and infected within the observation window.

### 3.4. Sensitivity Analyses

The sensitivity analyses are summarized in Supporting Information 1: Table S1 and Supporting Information 2: Figure S3. The estimated infectious period *r* was robust across all scenarios (posterior medians: 6.7–9.1 days). The ordering of the latent period across isolates, with Hongcheon/2023 being the shortest, was preserved in every scenario examined: under the day‐0 and day‐3 exposure assumptions, the Hongcheon/2023 latent period (medians 4.96 and 4.22 days, respectively) remained shorter than those of Paju1/2019 (8.48 and 8.06 days) and Yeongwol/2021 (8.20 and 6.94 days), with nonoverlapping 95% CrIs; the same held under equal distance weights. Under the common latent‐period prior, the Hongcheon/2023 estimate was essentially unchanged (median 3.90 days; 95% CrI 2.90–4.90, versus 3.77 days at the rerun baseline), indicating that the shorter latent period was not induced by its baseline prior. When the *E* compartment was excluded from the distance function, thereby removing any direct dependence of the fitting target on the assumed exposure time, the point‐estimate ordering was retained (Hongcheon/2023, 4.36 days; Yeongwol/2021, 6.54 days; Paju1/2019, 6.37 days), although the CrIs widened and overlapped, reflecting the reduced information content. In contrast, the absolute values of *β* and the SEIR‐derived *R*_0_ were sensitive to the assumed exposure time: assuming exposure at day 0 compressed the interval between exposure and the first transmission events and inflated *β* several‐fold (e.g., Paju1/2019, 1.83 versus 0.49 per pair per day at the rerun baseline). Accordingly, the absolute values of *β*, *ε*, and the SEIR *R*_0_ reported here should be interpreted as conditional on the exposure‐time reconstruction, whereas the relative comparisons among isolates, which share an identical assumption, and the infectious‐period estimates are robust.

## 4. Discussion

This study provides the first comparative analysis of within‐pen transmission dynamics among Korean ASFV isolates representing different phases of the Korean ASF epidemic (2019, 2021, and 2023). Using a Bayesian ABC‐SMC framework applied to a deterministic SEIR model together with a stochastic ABM, we estimated transmission coefficients, latent periods, infectious periods, and per‐donor secondary infection estimates for the three isolates. All three experiments resulted in complete spread (100% attack rate in DC pigs), consistent with the high within‐pen transmission potential of ASFV under experimental cohousing conditions.

The per‐donor secondary infection estimates (*R*_pd_) from the ABM (1.95–2.37) are not basic reproduction numbers and are therefore not directly comparable with published *R*_0_ values; nevertheless, their magnitude is of a similar order to within‐pen *R*_0_ estimates reported for ASFV genotype II in comparable experimental settings. Guinat et al. [15] reported an *R*_0_ of 2.5 (95% CI: 1.3–4.8) for the Georgia 2007/1 strain in a pen of 10 pigs using a stochastic SEIR model. Main et al. [16] reported within‐pen *R*_0_ values of 1.8–4.6 for European ASFV strains. Oh et al. [17] reported *R*_0_ estimates of 2.916 (exponential growth) and 4.015 (maximum likelihood) for a Vietnamese isolate. Our ABM estimates fall at the lower end of this range, which may partly reflect the small‐pen size (*n* = 5 DC pigs per experiment), a relatively short observation period, and the structural upper bound of 2.5 imposed on the per‐donor estimate by the experimental design (five DC pigs per two donors). Nevertheless, the lower 95% CrI bounds of the SEIR‐derived *R*_0_ exceeded 1 for all three isolates (Table 1), and the ABM PIs encompassed the observed infectious trajectories in all three experiments (Figure 4), confirming the capacity for efficient pen‐level spread characteristic of ASFV genotype II.

One of the most salient findings of this study is the markedly shorter posterior latent period (*ε*) estimated for the Hongcheon/2023 isolate (*ε* = 3.78 days; 95% CrI: 2.78–4.83) compared with those of the Paju1/2019 (*ε* = 7.23 days; 95% CrI: 5.36–9.48) and Yeongwol/2021 (*ε* = 6.11 days; 95% CrI: 4.57–7.73) isolates, with nonoverlapping 95% CrIs. This ordering was preserved across all sensitivity scenarios examined (Section 3.4; Supporting Information 1: Table S1; Supporting Information 2: Figure S3), and the finding is corroborated by the empirical data: DC pigs in group 3 (Hongcheon) first showed positive rectal swabs at 8.8 ± 0.8 dpi, ~1.6–2.8 days earlier than those in groups 1 and 2. A shorter latent period reduces the time window available for detection and intervention before infected pigs become contagious, which may translate into faster within‐herd spread in field settings. Nevertheless, because the absolute value of *ε* is conditional on the assumed exposure time and the CrIs overlapped those of the Yeongwol/2021 isolate under some alternative specifications (e.g., when the *E* compartment was excluded from the distance function), this finding should be regarded as a robust ordering rather than a precise quantification of the difference.

The shorter latent period observed for Hongcheon/2023 may reflect the differences in the intrinsic replication kinetics of this isolate. Because Hongcheon/2023 was recovered from a later phase of the Korean epidemic, it is possible that the virus accumulated mutations facilitating more rapid initial replication in monocytes and macrophages, potentially leading to earlier viremia and viral shedding in contact‐exposed pigs. Previous studies have shown that epidemiologically distinct ASFV isolates within the same genotype II clade can differ in virulence and replication efficiency [17, 24]. However, given the small number of DC pigs in each group (*n* = 5), these differences should be interpreted with caution, and a larger‐scale experiment would be required to confirm these temporal trends.

The within‐pen *R*_0_ values estimated from the Bayesian SEIR model (11.8–20.2) are higher than the ABM‐derived *R*_pd_ values (1.95–2.37) and those from comparable published studies. This difference is attributable to the different quantities estimated by the two approaches. The Bayesian SEIR *R*_0_ is calculated as *β* · *S*_0_ · *r* (with *S*_0_ = 5), which reflects the total expected number of secondary exposures generated by one infectious individual in a closed pen of five fully susceptible animals, where density‐dependent contact is assumed. In contrast, the ABM‐derived per‐donor estimate (*R*_pd_) represents the empirical mean number of DC pigs infected per inoculated donor, which is bounded by the pen size. Given that all five DC pigs were infected in all experiments, both approaches are consistent with near‐certain pen‐level spread; the SEIR *R*_0_ values simply reflect the high within‐pen contact rates inherent in the closed BSL‐3 housing conditions. This distinction between modeling‐framework‐specific *R*_0_ definitions and empirical per‐donor estimates such as *R*_pd_ is an important methodological consideration when comparing transmission parameters across studies [13, 25].

The main route of ASF transmission within pigsties is DC between infectious and susceptible pigs [15]. The three Korean ASFV isolates tested in this study were efficiently transmitted from inoculated donors to all directly cohoused pigs in each group within 18 days. These findings confirm that the DC route alone is sufficient to ensure a complete within‐pen spread for all three Korean isolates, regardless of the year of isolation. In a farm setting, additional routes, including fomite transmission, human movement, and indirect contact between adjacent pens, could further accelerate the spread, implying that within‐farm *R*_0_ values may substantially exceed the within‐pen estimates presented here.

Several limitations should be acknowledged. First, this study examined transmission dynamics under controlled BSL‐3 experimental conditions, where the pen size is necessarily small (*n* = 5 DC pigs per experiment) due to practical and ethical constraints. The small‐sample size results in a wide posterior CrI for all parameters, limiting the precision of interisolate comparisons. Second, the SEIR‐ODE model is a continuous deterministic approximation of a discrete stochastic process, which may not capture all sources of variability inherent in small‐pen dynamics. Third, all three experiments were conducted in a BSL‐3 facility with identical housing conditions, which may not fully represent the diverse environmental conditions present on commercial pig farms. Fourth, because each isolate was evaluated in a single experiment conducted in a different year (2019, 2021, and 2023), the isolate identity is completely confounded with the experiment year. Although the facility, housing conditions, inoculation dose, pig breed and age, and sampling protocols were kept identical across experiments, unmeasured factors that vary with time, such as animal sourcing, personnel proficiency, or subtle differences in study conduct, cannot be excluded and cannot be adjusted for analytically under the present design. The observed differences should therefore be interpreted as differences among experiments that are consistent with, but not conclusive proof of, intrinsic isolate‐specific transmission characteristics; confirming isolate effects would require contemporaneous head‐to‐head experiments with randomization. Fifth, all observations were collected at daily intervals, so infection and shedding‐onset times are known only within 1‐day window; the apparent precision of the posterior summaries should be interpreted in light of this interval censoring. Notwithstanding the limitations, this study is one of the very few attempts to quantitatively compare within‐pen ASFV transmission dynamics across Korean isolates from different epidemic phases using Bayesian and ABM approaches. Our finding that the Hongcheon/2023 isolate exhibits a shorter latent period than earlier Korean isolates may have practical implications for the design of ASF surveillance and early‐warning systems and warrants further investigation in larger‐scale transmission experiments.

## 5. Conclusions

This study estimates within‐pen transmission parameters for three Korean ASFV isolates (Korea/Pig/Paju1/2019, Korea/Pig/Yeongwol/2021, and Korea/Pig/Hongcheon/2023) using Bayesian ABC‐SMC SEIR modeling and stochastic ABM simulations. All three isolates produced 100% infection in cohoused DC pigs and yielded per‐donor secondary infection estimates (*R*_pd_) of ~2.0–2.4, consistent with the known high transmission efficiency of ASFV genotype II. The Hongcheon/2023 isolate is distinguished by a consistently shorter estimated latent period (*ε* ≈ 3.8 days), an ordering robust to alternative exposure‐time assumptions, distance‐function weightings, and prior specifications, and a higher simulated probability of complete pen‐level spread, suggesting potentially faster within‐pen viral spread than the 2019 and 2021 Korean isolates. Because isolate identity was confounded with the experiment year, this difference should be confirmed in contemporaneous head‐to‐head transmission experiments. These findings provide baseline transmission parameters for Korean ASFV that can inform mathematical models used to evaluate the control strategy effectiveness and highlight the importance of continued monitoring of Korean ASFV transmission characteristics as the epidemic evolves.

## Author Contributions

Da‐Young Kim and Ki‐Hyun Cho conducted the animal experiments and laboratory tests. Yong‐Myung Kang visualized the data. Ki‐Hyun Cho wrote the original draft. Dae Sung Yoo analyzed the data statistically, including Bayesian SEIR and agent‐based modeling, and reviewed and edited the manuscript. Yeon‐Hee Kim and Hae‐Eun Kang supervised the study.

## Funding

This work was supported by a grant from the Animal and Plant Quarantine Agency (Grant M‐1543085‐2025‐26‐01) from the Ministry of Agriculture, Food, and Rural Affairs, Republic of Korea.

## Disclosure

All authors have read and agreed to the published version of the manuscript.

## Ethics Statement

The animal experiments were approved by the Institutional Animal Ethics Committee of the Animal and Plant Quarantine Agency (Approval Numbers 2019‐499, 2021‐635, and 2023‐709) and were performed in accordance with the guidelines for the care and use of laboratory animals.

## Conflicts of Interest

The authors declare no conflicts of interest.

## Supporting Information

Additional supporting information can be found online in the Supporting Information section.

## Supporting information


**Supporting Information 1** Table S1: Sensitivity of Bayesian ABC‐SMC posterior estimates to alternative exposure‐time assumptions, distance‐function weightings, and prior specifications. Values are posterior medians with 95% credible intervals (CrIs). Baseline: direct‐contact pigs assumed exposed at day 4 (donor shedding onset), distance weights *E* = 1.0/*I* = 2.0/*R* = 1.5, baseline priors. Baseline values in this table were obtained from a separate rerun of the baseline specification performed together with the sensitivity scenarios so that all scenarios share a common reference; they therefore differ slightly from the primary estimates in Table 1 owing to Monte Carlo variability of the ABC‐SMC sampler. *R*_0_ SEIR = *β*·*S*_0_·*r* with *S*_0_ = 5.

**Supporting Information 2** Figure S1: Joint posterior scatter plots of *β* (*x*‐axis) versus *r* (*y*‐axis) for each isolate from Bayesian ABC‐SMC analysis. The dashed gray contour lines indicate *R*_0_ isocurves at 5, 10, 20, and 30. ABC‐SMC, approximate Bayesian computation sequential Monte Carlo. Figure S2: Distribution of the number of infected DC pigs per ABM simulation run (*n* = 2000) for each of three Korean ASFV isolates. The red dashed lines indicate the observed value (5/5 DC pigs infected in all experiments). Korea/Pig/Hongcheon/2023 isolate shows the highest probability of complete pen‐level spread. ABC‐SMC, approximate Bayesian computation sequential Monte Carlo. Figure S3: Sensitivity of posterior estimates of (A) the latent period (*ε*) and (B) the infectious period (*r*) to alternative model specifications for three Korean ASFV isolates. Points are posterior medians, and horizontal bars are 95% credible intervals from ABC‐SMC reruns under each scenario: baseline (DC exposure at day 4, distance weights *E* = 1.0/*I* = 2.0/*R* = 1.5, baseline priors); exposure of DC pigs assumed at day 0 (cohousing) or day 3 (donor viremia onset); exclusion of the *E* compartment from the distance function; equal compartment weights; vague uniform priors; and a common Gamma(4, 1) + 1 latent‐period prior for Hongcheon/2023. The Hongcheon/2023 isolate shows the shortest estimated latent period in every scenario, whereas infectious‐period estimates are robust throughout. ABC‐SMC, approximate Bayesian computation sequential Monte Carlo; ASFV, African swine fever virus; DC, direct contact.

## Data Availability

The data supporting the findings of this study are available from the corresponding author upon reasonable request. Analysis code (Python 3.10) and preprocessed SEIR state time series are available in the Supporting Information.
