# Supplementary material for: Evaluation of Transmission Parameters of Three Korean African Swine Fever Virus Isolates: A Bayesian ABC‐SMC and Agent‐Based Modeling Approach
Source: Transbound Emerg Dis. 2026 Nov 1;2026:4377003. doi: 10.1155/tbed/4377003 (PMC13725113; doi:10.1155/tbed/4377003)
Supplement: Supplementary file 1 [file TBED-2026-4377003-s002.docx]

**Figure S1.** Joint posterior scatter plots of β (*x*-axis) versus r (*y*-axis) for each isolate from Bayesian ABC-SMC analysis. The dashed gray contour lines indicate R₀ isocurves at 5, 10, 20, and 30. ABC-SMC, approximate Bayesian computation sequential Monte Carlo.

**Figure S2.** Distribution of the number of infected DC pigs per ABM simulation run (n = 2,000) for each of three Korean ASFV isolates. The red dashed lines indicate the observed value (5/5 DC pigs infected in all experiments). Korea/Pig/Hongcheon/2023 isolate shows the highest probability of complete pen-level spread. ABC-SMC, approximate Bayesian computation sequential Monte Carlo.

**Figure S3.** Sensitivity of posterior estimates of (A) the latent period (ε) and (B) the infectious period (r) to alternative model specifications for three Korean ASFV isolates. Points are posterior medians and horizontal bars are 95% credible intervals from ABC-SMC re-runs under each scenario: baseline (DC exposure at day 4, distance weights E = 1.0/I = 2.0/R = 1.5, baseline priors); exposure of DC pigs assumed at day 0 (co-housing) or day 3 (donor viremia onset); exclusion of the E compartment from the distance function; equal compartment weights; vague uniform priors; and a common Gamma(4, 1) + 1 latent-period prior for Hongcheon/2023. The Hongcheon/2023 isolate shows the shortest estimated latent period in every scenario, whereas infectious-period estimates are robust throughout. ABC-SMC, approximate Bayesian computation sequential Monte Carlo; ASFV, African swine fever virus; DC, direct-contact.
